# Using Coronary Guide Catheters with the Sheath-in-sheath Technique to Retrieve a Micra™ Leadless Pacemaker

**DOI:** 10.19102/icrm.2024.15052

**Published:** 2024-05-15

**Authors:** Hafez Golzarian, Wasim Rashid, Sandeep M. Patel, Mohammad Shaikh, Fayaz A. Hakim

**Affiliations:** 1 Internal Medicine Residency Program, Mercy Health St. Rita’s Medical Center, Lima, OH, USA; 2 Department of Interventional Cardiac Electrophysiology, Government Medical College Srinagar, Jammu and Kashmir, India; 3 Structural Heart & Intervention Center, Mercy Health—St. Rita’s Medical Center, Lima, OH, USA; 4 Heart Rhythm Services, Division of Cardiovascular Diseases, St. Rita’s Medical Center, Lima, OH, USA

**Keywords:** Amplatz coronary guide, Micra™ leadless pacing system, sheath-in-sheath, snare catheter retrieval

## Abstract

As the prevalence of leadless pacemaker systems increases, identifying various methodologies for retrieval of these devices in certain instances becomes even more paramount. We describe a case demonstrating the utility of a coronary guide catheter as part of an improvised sheath-in-sheath technique for the challenging retrieval of a Micra™ leadless pacing system (Medtronic, Minneapolis, MN, USA).

## Introduction

The Micra™ transcatheter leadless pacing system (LPS) (Medtronic, Minneapolis, MN, USA) is an attractive option for patients with an indication for single-chamber (right ventricular) pacing. Owing to its high implantation success rate and significantly lower complication rates compared to the transvenous pacing system, this device is being implanted with increasing frequency.^1,2^ The need for an early or late retrieval of LPS is rare. Although there are no dedicated tools for the retrieval of the Micra™ LPS, the currently used Micra™ delivery system and sheath-in-sheath techniques to snare and retrieve the device have been reported to be safe and effective.^3,4^ We describe a case to emphasize the utility of a coronary guide catheter as part of an improvised sheath-in-sheath technique for the acute retrieval of the Micra™ LSP.

## Case presentation

A 33-year-old woman with recurrent syncope was noted to have paroxysmal atrioventricular (AV) block with ventricular asystole on an event monitor during one of her syncopal episodes. She was otherwise healthy and active. Her physical examination was normal. Her echocardiogram, tilt table test, and neurological evaluation were unremarkable. She underwent a successful Medtronic Micra™ AV LPS implantation as per her preference over a conventional transvenous dual-chamber pacemaker. The device was successfully implanted in the right mid-interventricular septum using the technique described in the literature.^4^ Five thousand units of heparin bolus were administered intravenously soon after the introduction of the delivery sheath and repeated as needed to keep the activated clotting time at ≥250 s. The Micra™ introducer sheath was aspirated, flushed, and connected to a hepatized saline bag. Proper engagement of the device (3/4 tines) and stable device parameters (sensed R-waves, 11.7 mV; capture threshold, 0.88 V at 0.24 ms; and impedance, 696 Ω) were confirmed before the tether was removed. A pre-discharge device check 4 h post-implant showed abnormal device parameters with a sensed R-wave of 2.3 mV, a capture threshold of 5 V at 0.4 ms, and an impedance of 2000 Ω. A chest radiograph showed no change in the device location.

**Figure 1:**
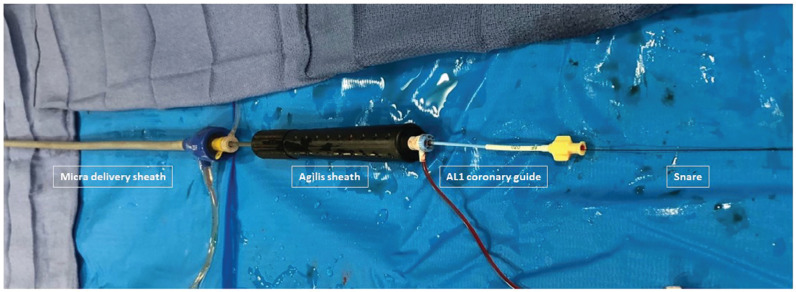
Working arrangement of the Micra™ introduction sheath, 11-French hemostatic sheath, Agilis sheath, and AL1 guide.

**Figure 2:**
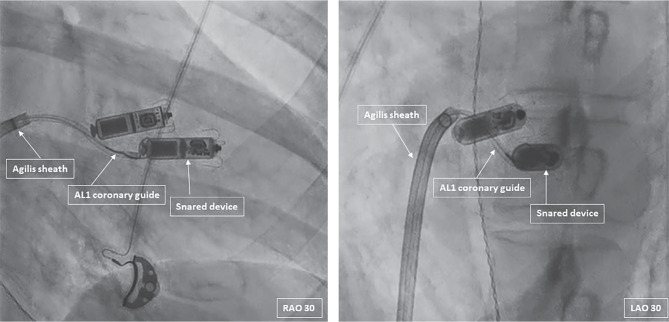
Fluoroscopic right and left anterior oblique views showing snaring of the malfunctioning device.

**Figure 3:**
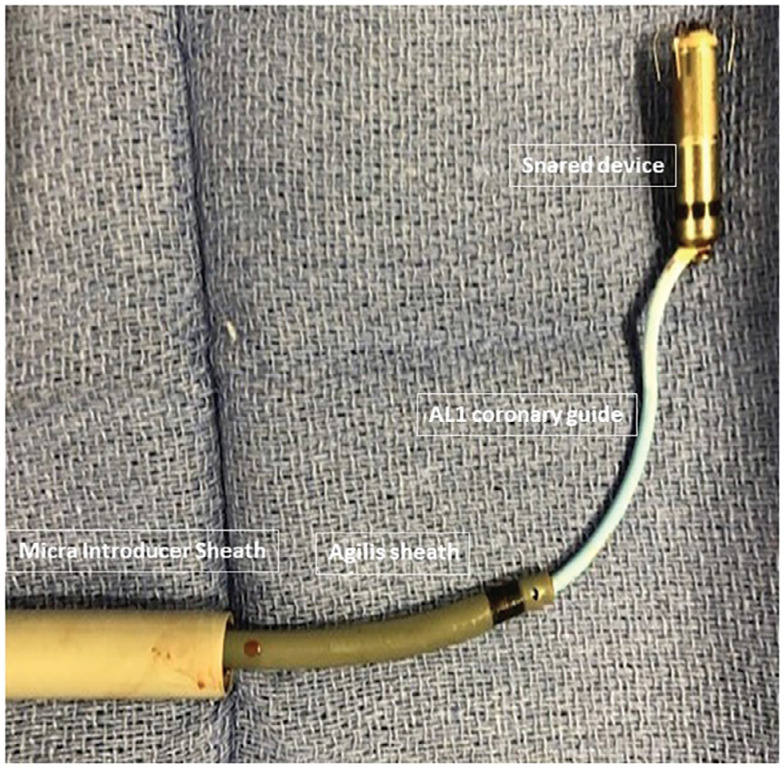
Retrieved Micra™ device and catheter arrangement.

Due to the persistent malfunctioning of the Micra™ device, a decision was made to retrieve it and implant a new system. The retrieval procedure was performed under deep sedation using a propofol infusion. The right femoral vein was cannulated under ultrasound guidance using a 21-gauge micropuncture needle, and a 0.035″ Amplatz stiff guidewire was advanced into the superior vena cava under fluoroscopic guidance. Following serial dilatation of the venous access site, using a 27-French (Fr) Micra™ introducer sheath and 23-Fr Micra™ delivery catheter complex, a new Micra™ AV LPS was implanted successfully on the right interventricular septum using the technique described in the literature. Intravenous heparin boluses were given to maintain the activated clotting time at ≥250 s. Leaving the Micra™ introducer sheath and Micra™ delivery catheter complex in place, a 200-cm-long, 7-mm single-loop snare-within-a-snare catheter was introduced through the Micra™ delivery catheter. Multiple attempts to snare the retrieval feature or the device itself were unsuccessful. We then decided to try the sheath-in-sheath technique. The Micra™ delivery catheter along with the snare/catheter was removed, and the Micra™ introducer sheath was aspirated and flushed with hepatized saline. An 11-Fr hemostatic short sheath was introduced into the Micra™ introducer sheath to prevent back bleeding, through which an 8.5-Fr, 91-cm-long large-curve deflectable sheath (Agilis; Abbott, Chicago, IL, USA) was introduced and advanced into the right ventricle over a 0.035″-long J-tip guidewire. A 120-cm-long, 10-mm single-loop snare was then introduced through the Agilis sheath. Again, multiple attempts to snare the retrieval feature or the device were unsuccessful. With both techniques, the snare fell short of the retrieval feature and buckled back every time an attempt to approximate the snare catheter with the proximal retrieval feature or to pass the snare over the device was made. We then exchanged the delivery catheter of the snare with a 100-cm 6-Fr Amplatz 1 coronary guide catheter (AL1, Cordis ADROIT, Milpitas, CA, USA) for better support and torque transmission **(Figure 1)**. This allowed us to approximate the AL1 coronary guide catheter with the proximal retrieval feature of the device and snare it without much difficulty **(Figure 2)**. The Agilis sheath was then advanced over the AL1 coronary guide toward the snared proximal retrieval feature. Continuous traction was applied to the snare to secure the grip over the proximal retrieval feature and to disengage the nitinol anchoring tines. Once released, the device was retrieved into the Micra™ introducer sheath **(Video 1)**. The Micra™ introducer sheath–Agilis sheath–AL1 coronary guide complex and the snared device were then removed **(Figure 3)**. A figure-of-eight suture was applied to the vascular access site to achieve hemostasis, and the patient was discharged home the following day. At the 6-month follow-up, the patient had had no syncopal event. The device parameters remained stable with <2% ventricular pacing.

Consent was obtained from the subject involved in this case study, and additional details are available from the authors upon request.

## Discussion

Unlike the Avier LPS (Abbott), there are no dedicated tools for the retrieval of the Medtronic Micra™ LPS. Despite limited experience, the two techniques that currently exist, which employ the Micra™ delivery catheter or a deflectable long sheath introduced through the Micra™ introducer sheath to snare the Micra™ LPS, have been reported to be safe and effective, even for devices with long dwell times.^5,6^ However, challenges in retrieving the Micra™ LPS may be encountered, especially in the case of device embolization that may necessitate modification or improvisation of the above-mentioned techniques. The use of a 6-Fr Amplatz (AL2; Cordis) diagnostic catheter has been previously reported to retrieve a dislodged Micra™ LPS within the right ventricle^7^ and an embolized device from the pulmonary artery.^8^ Recently, a successful retrieval of a Micra™ LPS with a dwell time of 5.5 years using the Avier retrieval catheters was reported in one case.^9^ We report a case where AL1 facilitated a successful acute retrieval of a non-displaced malfunctioning Micra™ LPS using the sheath-in-sheath technique in a difficult situation.

The catheters used to introduce snares are soft and hence may be difficult to maneuver. This, combined with a limited working length of the Agilis sheath, especially in an enlarged right ventricle, may pose a challenge to approximate and snare the proximal retrieval feature or even the device itself. A similar situation may be encountered if the device is behind or between thick trabeculae or if there is a clot formation over the proximal retrieval feature, which could have been a reason in our case. The use of an AL1 coronary guide to introduce a snare in such a situation may be a useful tool to maneuver the AL1 guide, along with placing the snare between the trabeculae or even dislodging the clot to compliment the sheath-in-sheath technique, as illustrated in our case. The coronary catheters and guides are relatively stiff and allow better control and torque transmission.

In a report of a worldwide registry of 40 Micra™ LPS retrievals, the most common reason for early retrieval was a significant increase in the capture threshold after tether removal.^4^ The most plausible explanation for the acute rise in the capture threshold requiring acute retrieval is clot formation at the tissue–device interface or inadequate engagement of the tines in the trabeculated tissue of the right ventricle. Hence, it is advisable to administer a bolus of heparin before or soon after the introduction of the Micra™ introducer sheath and then, as needed, tug on to the tether a few times and confirm engagement of at least 2/4 nitinol Flex tines before cutting the tether. LPS devices can be programmed in a non-functional mode and abandoned at end of life or device malfunction, and an additional device can be implanted adjacent to the non-functional device. However, this may not be an option in the case of device infection or embolization, where retrieval of the device is necessary. As the volume of implanted Micra™ LPS devices grows, an increasing need for early or late retrieval is expected to follow. Arguments for early retrieval after the tether is cut versus abandoning the device after inactivating it and implanting a new system in the case of post-implantation device malfunction are examples of more challenging extractions in the future if a compelling indication such as device infection emerges and there is a potential risk of tricuspid regurgitation with additional devices.^5^

## Conclusions

Use of an interventional Amplatz 1 coronary guide may complement the sheath-in-sheath technique for successful acute and possible late retrieval of a Micra™ LPS in some difficult scenarios.

## Figures and Tables

**Video 1:** Disengagement of the Nitinol tines and retrieval of the snared Micra™ device into the introducer sheath.
